# Allopregnanolone-induced rise in intracellular calcium in embryonic hippocampal neurons parallels their proliferative potential

**DOI:** 10.1186/1471-2202-9-S2-S11

**Published:** 2008-12-03

**Authors:** Jun Ming Wang, Roberta Diaz Brinton

**Affiliations:** 1 Department of Pharmacology and Pharmaceutical Sciences and Program in Neuroscience, University of Southern California, Los Angeles, CA 90089, USA; 2 Department of Pathology, University of Mississippi Medical Center, Jackson, MS 39216, USA

## Abstract

**Background:**

Factors that regulate intracellular calcium concentration are known to play a critical role in brain function and neural development, including neural plasticity and neurogenesis. We previously demonstrated that the neurosteroid allopregnanolone (APα; 5α-pregnan-3α-ol-20-one) promotes neural progenitor proliferation *in vitro *in cultures of rodent hippocampal and human cortical neural progenitors, and *in vivo *in triple transgenic Alzheimer's disease mice dentate gyrus. We also found that APα-induced proliferation of neural progenitors is abolished by a calcium channel blocker, nifedipine, indicating a calcium dependent mechanism for the proliferation.

**Methods:**

In the present study, we investigated the effect of APα on the regulation of intracellular calcium concentration in E18 rat hippocampal neurons using ratiometric Fura2-AM imaging.

**Results:**

Results indicate that APα rapidly increased intracellular calcium concentration in a dose-dependent and developmentally regulated manner, with an EC_50 _of 110 ± 15 nM and a maximal response occurring at three days *in vitro*. The stereoisomers 3β-hydroxy-5α-hydroxy-pregnan-20-one, and 3β-hydroxy-5β-hydroxy-pregnan-20-one, as well as progesterone, were without significant effect. APα-induced intracellular calcium concentration increase was not observed in calcium depleted medium and was blocked in the presence of the broad spectrum calcium channel blocker La^3+^, or the L-type calcium channel blocker nifedipine. Furthermore, the GABA_A _receptor blockers bicuculline and picrotoxin abolished APα-induced intracellular calcium concentration rise.

**Conclusion:**

Collectively, these data indicate that APα promotes a rapid, dose-dependent, stereo-specific, and developmentally regulated increase of intracellular calcium concentration in rat embryonic hippocampal neurons via a mechanism that requires both the GABA_A _receptor and L-type calcium channel. These data suggest that APα-induced intracellular calcium concentration increase serves as the initiation mechanism whereby APα promotes neurogenesis.

## Background

Allopregnanolone (APα; 3α-hydroxy-5α-hydroxy-pregnan-20-one; also known as tetrahydroprogesterone) is a derivative of progesterone that is produced in both the periphery and the central nervous system via enzymatic conversions of progesterone [1-3]. In mature neurons, APα is known to act as an allosteric modulator of the γ-aminobutyric acid type A (GABA_A_) receptor, binding to a specific site within the GABA_A _receptor at physiological concentrations (6–35 nM) [4,5] to increase chloride influx, thereby hyperpolarizing the neuronal membrane potential, and decreasing neuron excitability [6-11]. In marked contrast, the flux of chloride in developing neurons is opposite to that of mature neurons. Because of the high intracellular chloride content in immature neurons, APα provokes an efflux of chloride through the GABA_A _receptor, depolarization of the membrane, opening voltage dependent L-type calcium channels, leading to an influx of calcium from the extracellular medium [12-16].

Calcium signalling plays a key role in neural function and neural development [17-20]. Increases in intracellular calcium concentration ([Ca^2+^]_i_) also control cell cycle protein expression and promote cell proliferation [21-26]. Therefore, GABA_A _receptor-mediated depolarization may be the trigger that leads to activity-independent [Ca^2+^]_i _rise in early precursor cells, or neural progenitors and stem cells, and consequently may influence early developmental events, including neurogenesis and synaptogenesis [16,27-29].

Previously we found that APα rapidly induced neurite regression in cultured hippocampal neurons [30], which we later identified as a prelude to entry into the cell cycle and mitosis [29]. Recently, we demonstrated that APα regulates the expression of genes encoding cell cycle-related molecules and enhances human cortical neural progenitor, and rat hippocampal neuronal progenitor cell proliferation *in vitro *[29], and *in vivo *in triple transgenic Alzheimer's disease mice dentate gyrus [31-33]. Moreover, the L-type calcium blocker nifedipine abolished the APα-induced cell proliferation. We therefore hypothesized that the APα-induced neural progenitor cell proliferation is mediated by calcium influx via GABA_A _receptor-activated L-type calcium channels. To test this hypothesis, we investigated the impact of APα on calcium dynamics using Fura2 fluorescent ratio calcium imaging in rat E18 hippocampal neurons.

## Methods

### Animals and primary hippocampal neuron culture

Timed-pregnant Sprague-Dawley rats were purchased from Harlan Sprague Dawley, Inc. (Indianapolis, IN, USA). Rats were housed under controlled conditions of temperature (22°C), humidity (30–50%), and light (14 hour light:10 hour dark); water and food were available *ad libitum*. All experiments conformed to the Animal Welfare Act, Guide to Use and Care of Laboratory Animals, and the US Government Principles of the Utilization and Care of Vertebrate Animals Used in Testing, Research, and Training guidelines on the ethical use of animals. In addition, the minimal number of required animals was used for these experiments and suffering was minimized. Primary cultures of dissociated hippocampal neurons were performed as previously described [29,30]. Briefly, hippocampi were dissected from the brains of E18 rat fetuses, treated with 0.02% trypsin in Hank's balanced salt solution (HBSS, Invitrogen, Grand Island, NY, USA) for 5 minutes at 37°C and dissociated by repeated passage through a series of constricted, fire-polished Pasteur pipettes. Cells were plated onto poly-D-lysine-coated 22 mm diameter cover slips at a density of 2–4 × 10^4 ^cells per cm^2^, and grown in Neurobasal medium without phenol red (NBM; Gibco/Life Technologies, St. Petersburg, FL, USA) supplemented with 2% B27 (Gibco/Life Technologies), 10 U/ml penicillin, 10 μg/ml streptomycin, 0.5 mM glutamine and 25 μM glutamate. Cultures were maintained at 37°C in a humidified 5% CO_2 _atmosphere until the day of imaging.

### Steroids

All steroids used in this study were purchased from Steraloids.Inc (Newport, Rhode Island, USA). They were: allopregnanolone (APα; 5α-pregnan-3α-ol-20-one); pregnanolone (3α5βAP; 5β-pregnan-3α-ol-20-one); epipregnanolone (3β5βAP; 5β-pregnan-3β-ol-20-one); epiallopregnanolone (3β5αAP; 5α-pregnan-3β-ol-20-one); and progesterone (P_4_; 4-pregnane-3,20-dione).

### [Ca^2+^] microfluorimetry and imaging

[Ca^2+^] in hippocampal neurons was determined by ratiometric imaging of the Ca^2+^-sensitive fluorescent dye, Fura-2 acetooxymethyl ester (Fura-2 AM, Molecular Probes, Eugene, OR, USA), as previously described [34,35]. Briefly, cells were loaded with 2 μM Fura-2 AM in HEPES-buffered solution (HBS; 100 mM NaCl, 2.0 mM KCl, 1.0 mM CaCl_2_, 1.0 mM MgCl_2_, 1.0 mM NaH_2_PO_4_, 4.2 mM NaHCO_3_, 12.5 mM HEPES and 10.0 mM glucose, pH 7.4) for 45 minutes at 37°C, 5% CO_2_. Cells were then washed in HBS to remove excess Fura-2 AM and incubated in HBS for another 30 minutes to equilibrate. The cover slip with cells loaded with Fura-2 AM was then mounted in a perfusion chamber and placed on an inverted microscope (Olympus IMT-2). Baseline [Ca^2+^] was obtained over 1 minute prior to the initiation of stimulus, which was maintained for the duration of the imaging. Stimulation was initiated by perfusion (2 ml/minute), or bolus addition (200 μl in total of 1,000 μl), to a static bath of the indicated compounds. Fura-2 was successively excited by a xenon light source at 340 nm and 380 nm by means of two narrow beam band-pass filters selected by a computer-controlled filter wheel. The emitted fluorescence was filtered through a 520 nm filter, captured with an intensified CCD camera (COHU, San Diego, CA, USA), and analyzed with InCyt Im2 software (Intracellular Imaging, Inc., Cincinnati, OH, USA). The [Ca^2+^] was calculated by comparing the ratio of fluorescence at 340 nm and 380 nm against a standard curve. The curve was made from five different [Ca^2+^] standards available from Molecular Probes. Data from regions of interest were displayed in real-time and logged to hard disk. Data are presented as representative traces averaged from at least 10 cells per experiment. Responses to treatments were quantified by determining the difference between the average [Ca^2+^]_i _for the period of time of maximal response during the drug exposure and the average [Ca^2+^]_i _for 30 seconds prior to exposure. Changes in [Ca^2+^]_i _are presented as mean ± standard error of the mean (SEM) from three or more independent experiments with ≥10 cells per experiment. Statistical comparisons utilized one-way ANOVA followed by Newman-Keul's *post hoc *analysis.

## Results

### APα induces a rapid and transient change in [Ca^2+^]_i _in hippocampal neurons

**Figure 1:**
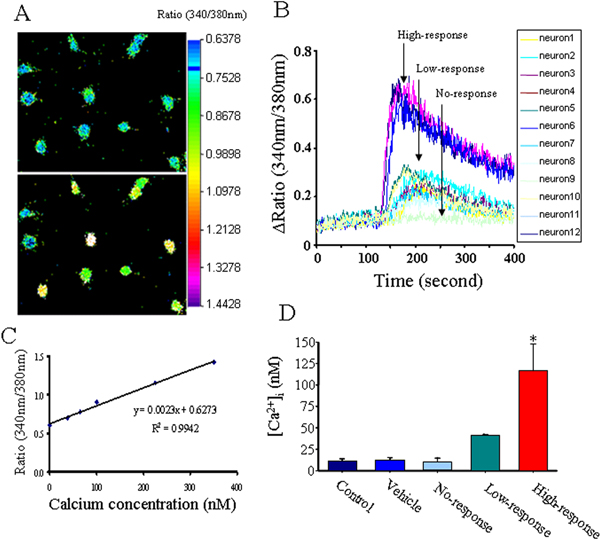
APα induces a rapid and transient [Ca^2+^]_i _rise in rat E18 hippocampal neurons in primary culture. Cells grown on glass cover slips were loaded with Fura-2 AM as described in the Methods. The [Ca^2+^]_i _was determined on single cells in HBSS medium. **(A) **Images represent calcium fluro-2 fluorescence in rat hippocampal neurons under vehicle control (top) and 500 nM APα (bottom). A fluorescent gradient of fluorescence ratio of 340 nm/380 nm is presented at the right side of the images. **(B) **A linear regression and the multiple correlation coefficient R-square value indicate the perfect correlation between fluorescence ratio of 340 nm/380 nm and [Ca^2+^]. **(C) **APα induced rapid and transient responses of [Ca^2+^]_i _recorded in a time course and expressed as the fluorescence ratio between 340 nm/380 nm, which is a representative of three different experiments. **(D) **Bar graph represents the summary of [Ca2+]_i _in response to control, vehicle control, and 500 nM APα. Data are mean ± SEM (*p < 0.05 compared to control neurons; N = 3 experiments with 36 neurons/condition/experiment). No changes in [Ca^2+^]_i _were observed under control or vehicle control conditions. With the addition of 500 nM APα, three types of calcium responses were observed in neurons: high (defined as an increase in [Ca^2+^]_i _>65 nM over the baseline); low (defined as an increase in [Ca^2+^]_i _<45 nM over the baseline); and no response (defined as neurons that did not show a measurable increase in [Ca^2+^]_i _over their baseline).

APα induced a rapid (within seconds) and transient (over the course of minutes) increase in [Ca^2+^]_i _in E18 rat hippocampal neurons (Figure 1A) as observed by ratiometric imaging of the Ca^2+^-sensitive fluorescent dye Fura-2 AM (Figure 1A). [Ca^2+^]_i _was calculated using a linear regression standard curve of the 340 nm/380 nm ratios of fluorescence generated by a standard series of calcium concentrations (Figure 1B). The multiple correlation coefficient (R-square = 0.9942) indicated a linear relationship between fluorescence ratio and [Ca^2+^]_i _(Figure 1B). Based on this linear relationship, it was possible to generate an approximate [Ca^2+^]_i _value. Three distinct 'calcium responses' in neurons exposed to APα emerged from this analysis: high response, neurons exhibiting a ≥0.78 of 340 nm/380 nm ratio; low response, neurons exhibiting ≤0.73 of 340 nm/380 nm ratio; and no response (Figure 1C). In the low response population, APα (250 nM) induced a [Ca^2+^]_i _rise of 38 ± 2.6 nM, whereas in the high response population, the same concentration of APα induced a [Ca^2+^]_i _rise of 118 ± 32 nM (Figure 1D; *p *< 0.05 compared to control neurons).

### APα-induced [Ca^2+^]_i _rise is dependent upon dose and days *in vitro *and is stereoisomer specific

**Figure 2:**
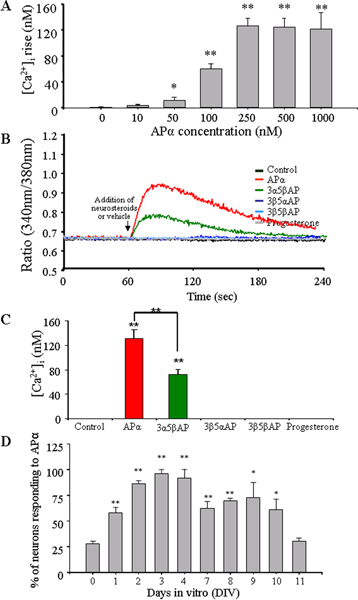
APα-induced [Ca^2+^]_i _rise shows dose- and DIV-dependent manner and is stereoisomer-specific. **(A) **Bar graph shows dose responses of APα on [Ca^2+^]_i _rise in 3 DIV neurons. Data are mean ± SEM (N = 3–6 experiments with 36 neurons/condition/experiment, *p < 0.05, **p < 0.01). **(B) **Stereospecificity of APα-induced [Ca2+]_i_. Neurons were exposed to 250 nM of the stereoisomers APα, 3α5βAP, 3β5αAP, 3β5βAP or progesterone (250 nM) at the time-point indicated by the arrow. The graph is a representative of four different experiments (N = 36 neurons/condition/experiment). **(C) **Bar graph shows the summary of data collected from stereoisomer experiments and represented as [Ca^2+^]_i_. **p < 0.01 APα or 3α5βAP compared to control, or APα and 3α5βAP compared to each other; N = 4 experiments with 36 neurons/condition/experiment. **(D) **Bar graph indicates the percentage of neurons that exhibit a [Ca^2+^]_i _rise in response to 500 nM APα at different days *in vitro *(DIV). DIV 0 is defined as the day the neurons are seeded. The greatest response, around 90% of cells responding to APα, is observed on 3 DIV. Data are mean ± SEM (N = 3 independent experiments with at least 36 neurons/condition/experiment). *p < 0.01, **p < 0.001 versus DIV 0.

To characterize APα-induced [Ca^2+^]_i _signalling in embryonic hippocampal neurons, three issues were addressed: dose-response; stereospecificity; and developmental profile of response during days *in vitro *(DIV). Analysis for dose-response and stereospecificity were conducted in neurons at 3 DIV. Application of 10 nM APα showed an insignificant increase in [Ca^2+^]_i_. A linear and significant increase was observed from 50 nM, (13 ± 7 nM [Ca^2+^]_i_, *p *< 0.05 versus vehicle control), and 100 nM APα (62 ± 12 [Ca^2+^]_i_, *p *< 0.01)_. _Maximal response was observed when 250 nM APα was applied (135 ± 14 nM [Ca^2+^]_i_, *p *< 0.01), which could not be further increased using 500–1,000 nM APα (Figure 2A). The estimated EC_50 _value for APα was 124 ± 15 nM.

To determine the specificity of APα on the increase of [Ca^2+^]_i_, the effects of several APα isomers, and its parent molecule, progesterone, were compared at a concentration of 250 nM. APα induced an [Ca^2+^]_i _increase with an average of 133 ± 11 nM. No significant [Ca^2+^]_i _response was observed for 3β5αAP, 3β5βAP, or progesterone at the same concentration. Only 3α5βAP induced a significant, but lower, [Ca^2+^]_i _rise (73 ± 8 nM, *p *< 0.01; Figure 2B,C). These data indicate that the APα-induced [Ca^2+^]_i _increase is sterospecific for APα, and its 5β-, but not 3β-isomers.

To determine the proportion of hippocampal neurons responsive to APα, and the duration of responsiveness, the number of APα-induced [Ca^2+^]_i_-responsive neurons were assessed each day for 11 days (216 neurons/day). The percentage of APα-induced [Ca^2+^]_i _rise in neurons increased linearly from day 0 to day 4 (DIV), followed by a stepwise decrease in the number of neurons that, by day 11, had returned to day 0 levels (Figure 2D). At DIV 0, 25 ± 5% of the neurons exhibited a APα-induced [Ca^2+^]_i _increase; at DIV 1, 61 ± 7%; at DIV 2, 84 ± 4%. By DIV 3, the number of APα-responding neurons reached an asymptote at 90 ± 6% (Figure 2D; *p < 0.01 and **p < 0.001) and remained at that level until DIV 4. From DIV 7–10, the number of responding neurons declined and remained stable at approximately 63%. By DIV 11, the number of neurons responding to APα (30 ± 4%) declined to the level observed at DIV 0.

### Different types of [Ca^2+^]_i _responses

**Figure 3:**
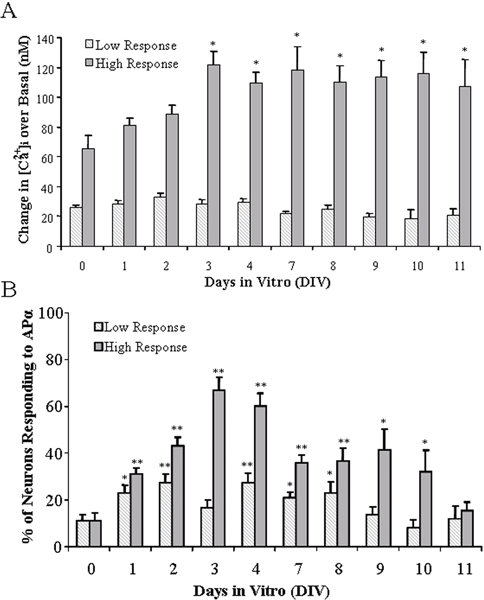
The magnitude of the APα-induced intracellular calcium response changes at different DIV. **(A) **Bar graph indicates the magnitude of the [Ca^2+^]_i _increases, high response (defined as >65 nM [Ca^2+^]_i _rise over the baseline) or low response (defined as <45 nM [Ca^2+^]_i _rise over baseline), in [Ca^2+^]_i _over the baseline in hippocampal neuron cultures at different DIV treated with 500 nM APα. **(B) **Bar graph indicates the percentage of hippocampal neurons that respond to 500 nM APα with a high or a low response at different DIV. Data are mean ± SEM (N = 3 independent experiments with at least 36 neurons/condition/experiment). *p < 0.01, **p < 0.001 versus DIV 0.

To determine the developmental profile for low and high responders, we determined the change in the magnitude of APα-induced [Ca^2+^]_i _in low and high responders, and the portion of the population of responders across DIV. We first investigated whether the magnitude of the APα-induced [Ca^2+^]_i _changed with time in culture. High-response, low-response, and non-responsive neurons were consistently observed from DIV 0 to DIV 11. As summarized in Figure 3A, the magnitude of APα-induced [Ca^2+^]_i _in low responders (340/380 nm fluorescence ratio lower than 0.73 and reflect <45 nM increase in free calcium; Figure 1B) showed no significant change during the entire 11-day experimental period. In contrast, the magnitude of APα-induced [Ca^2+^]_i _in high-response neurons (340/380 nm fluorescent ratio >0.78, reflecting a >65 nM increase in free calcium, *p *< 0.01) increased from 65 to 125 ± 10 nM in the first 3 days, and reached a plateau for the remaining days of culture. We then determined whether the number of high-response neurons versus low-response neurons changed during time in culture and present the data as a percentage of total cells (Figure 3B; *p < 0.01 and **p < 0.001). The number of high-response neurons increased rapidly and linearly, and reached the highest percentage of 65 ± 7% on DIV 3, gradually decreasing to less than 40% on DIV 7. The number of low-response neurons also increased early in the course of culturing and reached the greatest percentage of 27 ± 5% on DIV 2, decreasing to 17 ± 3% on DIV 3 (no significant difference with DIV 0). However, on DIV 4, the percentage of low-response neurons returned to a level similar to that observed on DIV 2 and then gradually decreased. DIV 3 was the time-point at which the highest number of high-response neurons, and the lowest number of low-response neurons, were observed. These data indicate that the magnitude of calcium responses, and the numbers of APα-induced [Ca^2+^]_i_-responsive neurons, vary with the days of culture and that DIV 3 represents a pivotal time-point in the response to APα.

### APα-induced [Ca^2+^]_i _rise reflects an influx of extracellular Ca^2+ ^and is regulated by an L-type calcium channel

**Figure 4:**
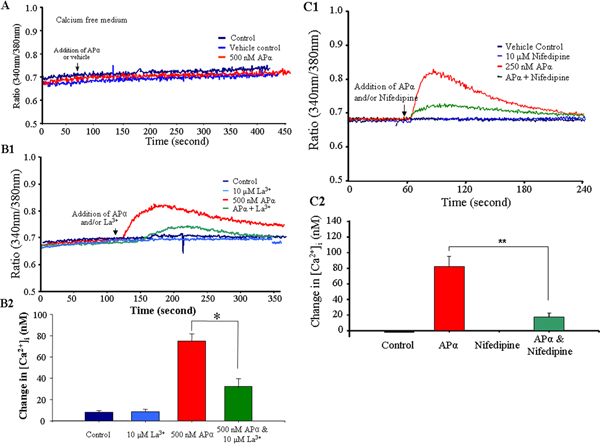
APα-induced [Ca^2+^]_i _increases result from influx of extracellular calcium and are mediated by the L-type calcium channel. **(A) **Neurons (3–4 DIV) were exposed to 500 nM APα or vehicle control in Ca^2+^-free medium at the time-point indicated by the arrow. In the medium without calcium, APα showed no effect on intracellular calcium concentration. A representative graph of four independent experiments is shown (N = 36 neurons/condition/experiment). **(B) **La^3+ ^diminished APα-induced [Ca^2+^]_i _rises. Neurons (3–4 DIV) were incubated with or without 10 μM La^3+ ^(a calcium channel blocker) for 30 minutes prior to and continuously throughout imaging, and 500 nM APα or vehicle was added at the time-point indicated by the arrow. **(B1) **Representative graph of three different experiments (N = 36 neurons/condition/experiment). **(B2) **Bar graph represents the summary of [Ca^2+^]_i _rise in response to control, 10 μM La^3+^, 500 nM APα, and APα + La^3+^. Data are mean ± SEM, *p < 0.05 compared to APα treated neurons; N = 3 experiments with 36 neurons/condition/experiment. **(C) **Nifedipine blocked APα induced [Ca^2+^]_i _rises. Neurons were incubated with or without 10 μM nifedipine (an L-type calcium channel blocker) for 30 minutes prior to and continuously throughout imaging, and 250 nM APα or vehicle was added at the time-point indicated by the arrow. **(C1) **The graph is representative of three different experiments (N = 36 neurons/condition/experiment). **(C2) **The bar graph represents quantitative changes in [Ca^2+^]_i _in response to control, 10 μM nifedipine, APα, and APα + 10 μM nifedipine. Data are mean ± SEM (**p < 0.01 compared to APα treated neurons; N = 36 neurons/condition/experiment).

Free [Ca^2+^]_i _increase could result from either an influx of extracellular calcium or a release of calcium from an intracellular pool stored in organelles, including the endoplasmic reticulum and mitochondria. To investigate whether the APα-induced [Ca^2+^]_i _rise required an influx of extracellular Ca^2+^, neurons at DIV 3–4, when 84% of neurons should be responsive to APα (65% high responders), were treated with 500 nM APα in a calcium-free medium. To sustain comparable osmolarity and ionic strength, 2 mM NaCl was substituted for 1 mM CaCl_2_. In the absence of extracellular calcium, APα did not induce a rise in [Ca^2+^]_i _(Figure 4A). This result suggested that the APα-induced [Ca^2+^]_i _rise could be attributed mainly, if not completely, to the influx of extracellular Ca^2+^. To discern which calcium channel was required for the calcium influx, neurons were exposed to the inorganic-calcium transport inhibitor La^3+ ^(10 μM) [36,37]. Exposure to La^3+ ^reduced the APα-induced [Ca^2+^]_i _rise from 78 ± 5 nM to 42 ± 7 nM (Figure 4B; *p *< 0.05). When nifedipine, a more specific blocker to L-type calcium channels was used, the APα-induced [Ca^2+^]_i _rise decreased from 82 ± 14 nM to 20 ± 4 nM (Figure 4C; *p *< 0.01). These data indicate that the APα-induced [Ca^2+^]_i _rise is dependent upon extracellular Ca^2+ ^and is regulated by the L-type calcium channel.

### GABA_A _receptor mediates the [Ca^2+^]_i _rise induced by APα

**Figure 5:**
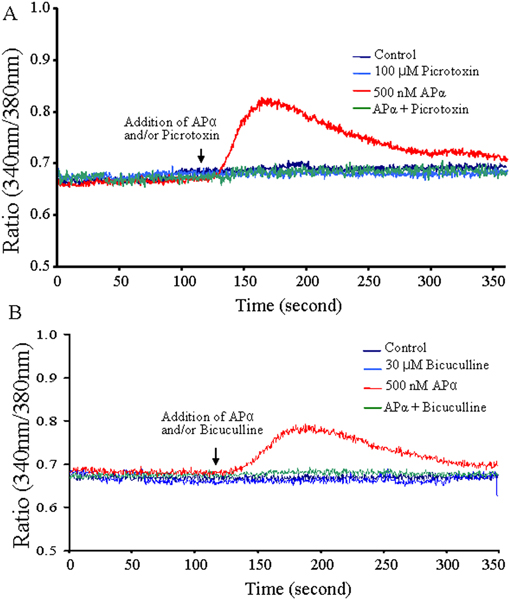
APα-induced [Ca^2+^]_i _rises are mediated by the GABA_A _receptor. **(A) **Picrotoxin completely abolished APα-induced [Ca^2+^]_i _rises. Fluorescent Fura2-AM imaging of 3 DIV hippocampal neurons incubated with or without 100 mM picrotoxin (a non-competitive GABA_A _receptor antagonist) 30 minutes prior to and continuously throughout imaging. APα (500 nM) or vehicle was added at the time-point indicated by the arrow. The graph is representative of the four experiments conducted (N = 36 neurons/condition/experiment). **(B) **Bicuculline completely abolished APα-induced [Ca^2+^]_i _rises. Fluorescent Fura2-AM imaging of 3 DIV hippocampal neurons incubated with or without 30 μM bicuculline (a competitive GABA_A _receptor antagonist) for 30 minutes prior to and continuously throughout imaging. APα (500 nM) or vehicle was added at the time-point indicated by the arrow. The graph is representative of the four different experiments conducted (N = 36 neurons/condition/experiment).

It is well known that APα acts as an allosteric modulator of the GABA_A _receptor/chloride channel by altering chloride flux and thereby hyperpolarizing (in mature neurons) or depolarizing (in immature neurons) the neuronal membrane [15,16,38-42]. To determine whether the APα-induced [Ca^2+^]_i _rise in hippocampal neurons required the GABA_A _receptor, two GABA_A _receptor blockers, picrotoxin (a non-competitive antagonist) and bicuculline (the prototypical competitive antagonist that directly competes with GABA for binding to the receptor complex), were applied at 100 μM and 30 μM, respectively. Both GABA_A_receptor blockers completely abolished the APα-induced [Ca^2+^]_i _rise (Figure 5A,B). These data indicate that the APα-induced [Ca^2+^]_i _rise requires activation of GABA_A _receptor and the L-type calcium channel in rat hippocampal neuron cultures.

## Discussion

Analyses using intracellular ratiometric Fura2 calcium imaging demonstrated that APα specifically induced a rapid, transient, and dose-dependent [Ca^2+^]_i _rise in E18 rat hippocampal neurons in primary culture. The APα-induced [Ca^2+^]_i _rise was not observed in calcium-free medium and was blocked by the L-type calcium channel blockers La^3+ ^and nifedipine. In addition, the GABA_A _receptor inhibitors picrotoxin and bicuculline completely abolished the APα-induced [Ca^2+^]_i _rise. These findings in hippocampal neurons parallel those described by Dayanithi and Tapia-Arancibia [43] in fetal rat hypothalamic neurons, suggesting that the APα-induced rise in [Ca^2+^]_i _is a generalized effect in developing neurons.

The APα-induced rapid response in [Ca^2+^]_i _rise was specific to APα as the β-isomers of APα, namely 3β5βAP and 3β5αAP, and its parent molecule, progesterone, were without effect, whereas 3α5βAP induced a [Ca^2+^]_i _rise that was 50% of the effect of APα. These data suggest the structure with the most important impact on stereospecificity may reside at the 3 position of the neurosteroid. Indeed, both an extracellular application, and 3α-substitutions of the A ring of the steroid nucleus, have been reported to be prerequisites for many of the interactions of the neurosteroid with GABA_A _receptor/Cl^- ^channels, because neither intracellular applications, nor 3β-substituted steroids applied extracellularly, show agonistic effects of GABA [4,44,45]. Recent analyses by Hosie *et al. *[10] indicate that APα can bind to two sites on the GABA_A _receptor, one that potentiates, and one that directly activates the GABA_A _receptor. The potentiating binding site of APα resides in a cavity formed by the α-subunit transmembrane domains. The direct activating binding site of APα located among interfacial residues between the α and β subunits and is enhanced by steroid binding to the potentiation site [10]. Data presented here demonstrate that the APα-induced [Ca^2+^]_i _rise can be abolished by two GABA_A _receptor blockers, namely bicuculline [46-49] and picrotoxin [50-52], strongly supporting the notion that the APα-induced [Ca^2+^]_i _rise is a GABA_A _receptor-mediated process and most likely through the direct activating binding site.

GABA_A _receptor is an ion channel that allows either influx or efflux of chloride ions (Cl^-^), depending upon the prevailing transmembrane [Cl^-^] gradient. Because immature neurons have a higher intracellular [Cl^-^], activation of the GABA_A _receptor by GABA, or other agonists, for example, muscimol, causes efflux of Cl^- ^and thus membrane depolarization [15,53]. This depolarization is sufficient to open L-type voltage-gated calcium channels, leading to calcium influx [54-56]. GABA and GABA-induced calcium influx have been linked to trophic actions important for developmental processes, including the expression of brain-derived neurotrophic factor [20]. Thus, GABA_A _receptor-mediated depolarization may be the trigger for a spontaneous, activity-independent [Ca^2+^]_i _rise in early precursor cells, or subventricular zone radial precursor cells, thereby influencing early developmental events, including neurogenesis and synaptogenesis [16,28,29]. In the present study, we have demonstrated that the APα-induced [Ca^2+^]_i _rise can be blocked by a voltage-gated calcium channel blocker, La^3+^, as well as a more specific L-type calcium channel blocker, nifedipine. Further, we recently demonstrated that the L-type calcium channel blocker nifedipine specifically abolished the APα-induced proliferation of rat hippocampal neuronal progenitor cells [29]. Taken together, these data suggest that the APα-induced [Ca^2+^]_i _rise, regulated by the L-type calcium channel and evoked by GABA_A _receptor, may be the signalling initiation mechanism for APα-induced neuroprogenitor cell proliferation and cell cycle gene expression.

As development progresses, the effect of APα associated with GABA_A _receptor binding gradually switches from excitatory to inhibitory [15,16,57,58]. The timing of the shift from depolarizing to hyperpolarizing via GABA_A _receptor varies across brain regions, but is generally complete by the second week of life in the rat and mouse [12,59-61]. Remarkably, APα induction of [Ca^2+^]_i _rise in cultured hippocampal neurons closely parallels the developmental time course of Na^+^-K^+^-2Cl^- ^co-transporter expression [62,63]. In primary cultures of the E18 rat hippocampal neurons, a mixture of E16–E18 neurons with differing phenotypes will exist. Therefore, it is not surprising that high and low APα-induced calcium responses were observed in this study. The high and low calcium responses may be indicative of the developmental stage of the neuron in culture.

The GABA_A _receptor is composed of a pentamer of structurally homologous subunits that may be drawn from the α1–6, β1–3, γ1–3, δ, ε, θ, ρ1–3, and π subunit families. The precise subunit composition of different GABA_A _receptor isoforms is an important determinant of their pharmacological and biophysical properties [4,64-69], but the exact combination heterogeneity exist. For example, a study indicated that the subunit combinations comprising α1β1γ2 and α3β1γ2 required a three- to seven-fold lower concentration for APα to enhance GABA-evoked current to the same degree as other combinations [70], while another study demonstrated that the potencies of APα to enhance GABA response were significantly higher in the α5β2γ2 receptor versus α1β2γ2 [71]. Another study suggested that the efficacy of APα to enhance GABA response depended on the γ subunit subtype: α1β1γ3 > α1β1γ2 = α1β1γ1 [72]. Interestingly, during migration from the subventricular zone to the cortical plate, neurons became predominantly GABAergic, and their dominant GABA_A _receptor subunit expression pattern changed from α4β1γ1 to α3β3γ2 or α3β3γ3, coincident with an increasing potency of GABA on GABA_A _receptor-mediated depolarization [73]. Although heterogeneity of the subunit combination exists, all these studies suggest that the effects of APα depend on subunit combinations of the GABA_A _receptor and the combination is changing during development. Therefore, the different GABA_A _receptor subunit combinations might be the underlying mechanism for the variety of APα-induced [Ca^2+^]_i _responses in cultured hippocampal neurons. Along with DIV, the combination of the GABA_A _receptor subunits is changing and a possible time-point at which one GABA_A _receptor subunit combination switches to another is 3–4 DIV. However, exact matching of neuronal high and low calcium responses to the expression of specific GABA_A _receptor subunit combinations, and investigation of the role of each combination, needs to be further addressed.

## Conclusion

These data demonstrate that APα induced a dose and developmentally regulated rise in [Ca^2+^]_i _that was stereospecific, rapid, and transient in E18 rat hippocampal neurons in primary culture. Moreover, our data indicated that the APα-induced rise in [Ca^2+^]_i_requires both GABA_A _receptor and L-type calcium channel. Together with earlier findings, we propose that the APα-induced rise in [Ca^2+^]_i _provides a mechanism whereby APα can promote proliferation of rodent, and also possible of human, neural progenitor cells.

## List of abbreviations used

3α5βAP: pregnanolone; 3β5αAP: epiallopregnanolone; 3β5βAP: epipregnanolone; APα: allopregnanolone; [Ca^2+^]_i_: intracellular calcium concentration; DIV: days *in vitro*; Fura-2 AM: Fura-2 acetooxymethyl ester; GABA_A_: γ-aminobutyric acid type A; HBS: HEPES-buffered solution; HBSS: Hank's balanced salt solution; SEM: standard error of the mean.

## Competing interests

The authors declare that they have no competing interests.

## Authors' contributions

JMW analyzed the data and drafted the manuscript. RDB designed the experiments, coordinated the study and edited the manuscript.
